# Applications of Genome-Scale Metabolic Models in Biotechnology and Systems Medicine

**DOI:** 10.3389/fphys.2015.00413

**Published:** 2016-01-07

**Authors:** Cheng Zhang, Qiang Hua

**Affiliations:** ^1^State Key Laboratory of Bioreactor Engineering, East China University of Science and TechnologyShanghai, China; ^2^Shanghai Collaborative Innovation Center for Biomanufacturing TechnologyShanghai, China

**Keywords:** genome-scale metabolic models, systems biology, metabolic capability analysis, *in silico* metabolic engineering, systems medicine

## Abstract

Genome-scale metabolic models (GEMs) have become a popular tool for systems biology, and they have been used in many fields such as industrial biotechnology and systems medicine. Since more and more studies are being conducted using GEMs, they have recently received considerable attention. In this review, we introduce the basic concept of GEMs and provide an overview of their applications in biotechnology, systems medicine, and some other fields. In addition, we describe the general principle of the applications and analyses built on GEMs. The purpose of this review is to introduce the application of GEMs in biological analysis and to promote its wider use by biologists.

## Introduction

Genome-scale metabolic models (GEMs) are reconstructions of the metabolic networks of many kinds of cells, including those of microorganisms, plants, and mammals. In some cases, GEMs could represent the whole tissue or body of a multicellular organism. In these metabolic networks, the gene-protein-reaction (GPR) relationships are annotated. In addition, all the reactions in GEMs are mass- and energy-balanced, ensuring stoichiometric balance. Thus, GEMs enable researchers to conduct system-level metabolic response analysis and flux simulation, which is not possible using general metabolic pathway databases such as KEGG. Furthermore, since GPR relationships are included in GEMs, other omics data such as transcriptomic and proteomic data could be systematically integrated into GEMs. Thus, GEM-based multi-omic analyses are more informative with stoichiometric balance and could possibly provide deeper biological insights.

In the past 15 years, GEMs have garnered considerable research attention. In 2000, the first GEM, a model of *Escherichia coli* MG1655, was reported (Edwards and Palsson, 2000). A few years later, a yeast GEM was published (Doerks et al., 2002), thus initiating a new era for systems biology. In the beginning, researchers tried to use GEM-based *in silico* simulations to guide the rational design of industrial microorganisms (hereafter referred to as *in silico* metabolic engineering). In 2003, a method called OptKnock (Burgard et al., 2003) was published and it employed a bi-level optimization program to search for reaction knockout targets that would yield overproduction of a desired biochemical while maintaining optimal growth. Following that, a series of *in silico* metabolic engineering methods were developed for various gene manipulations other than knock-out (Pharkya et al., 2004; Pharkya and Maranas, 2006; Choi et al., 2010; Ranganathan et al., 2010; Park et al., 2012; Chowdhury et al., 2014; Mahalik et al., 2014), leading to a marked expansion in the usage of GEMs. Furthermore, many of the *in silico* metabolic engineering methods were experimentally validated (Fong et al., 2005; Izallalen et al., 2008; Asadollahi et al., 2009; Brochado et al., 2010; Choi et al., 2010; Yim et al., 2011; Xu et al., 2011; Park et al., 2012; Ranganathan et al., 2012; Otero et al., 2013; Kim et al., 2014), which showed the power of GEM-based applications. With the development of systems biology, GEMs were also used as scaffolds for systematic integration of omics data because GEMs could be used to reconstruct the relationship among genes, enzymes, and metabolism. Numerous algorithms have been developed to integrate various types of omics data such as thermodynamics (Henry et al., 2007), transcriptomics/proteomics (Becker and Palsson, 2008; Colijn et al., 2009; Zur et al., 2010), fluxomics (Wiback et al., 2004), and metabolomics (Cakir et al., 2006). In return, the integration of omics data could improve the prediction of GEMs. More recently, GEM has been applied to systems medicine. Since the reconstruction of the first global GEM for humans, Recon 1, which was established in 2007 (Duarte et al., 2007), researchers have started to explore the possibility of clinical applications of GEMs and have reported several successful cases (Agren et al., 2014; Gatto et al., 2014; Jerby-Arnon et al., 2014). In fact, GEMs and their applications have received considerable attention recently.

Although GEMs are becoming increasingly popular, they are not easy to understand or use by non-experts. The complex code and script usually used for GEM-based computational applications and analyses are not readily available to the community of biologists, greatly hampering the wide usage of GEMs. In this review, we describe the key concepts and assumptions of GEMs. In addition, we describe the general principle of the applications and analyses built on GEMs. The information presented here is expected to promote the spread of GEM usage by biologists.

## Basic concept of GEMs

**Figure 1:**
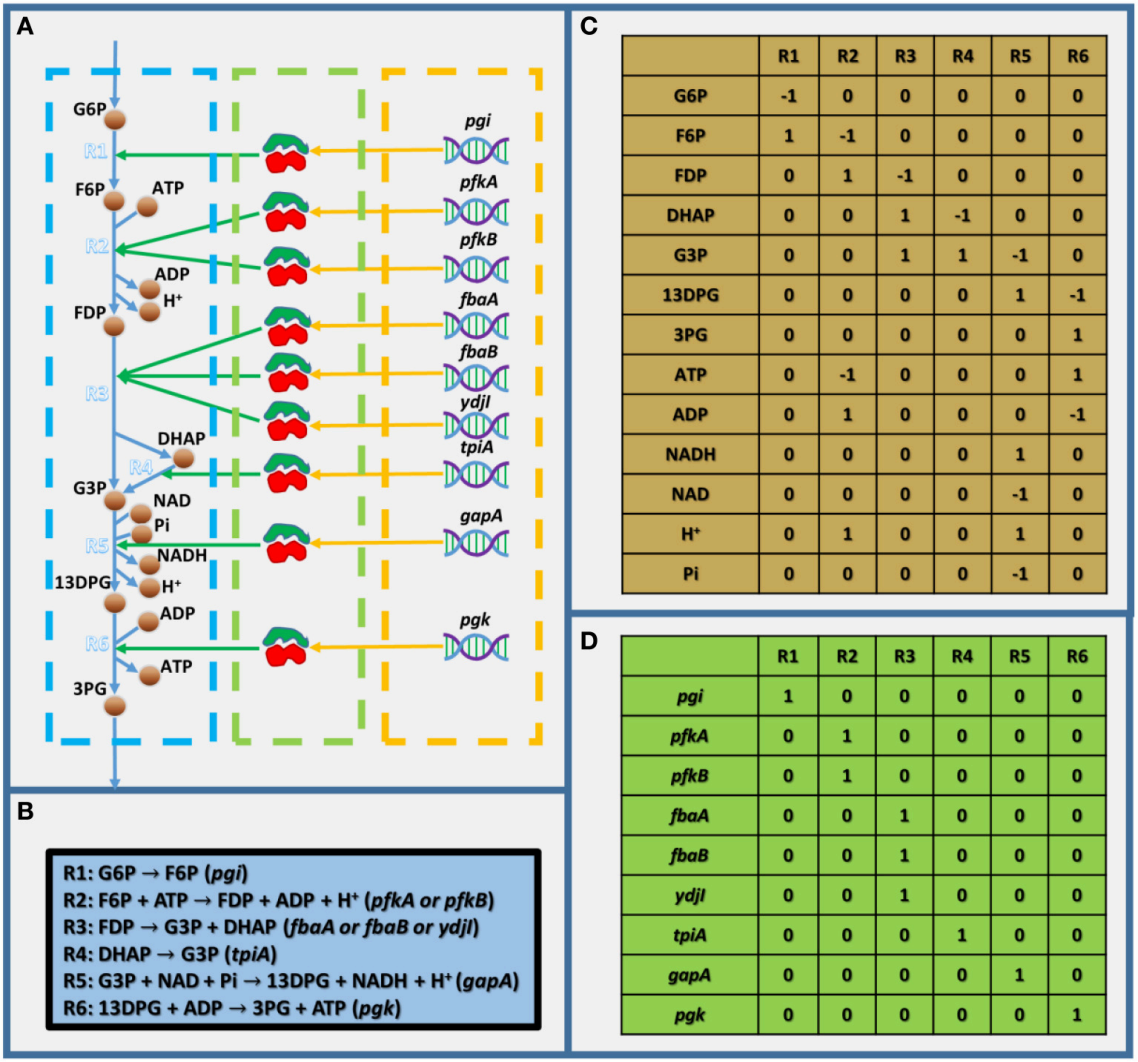
**Toy model showing the basic structure of GEMs**. **(A)** Visualized toy model,**(B)** biochemical equations within the toy model, **(C)** stoichiometric matrix of the toy model, and **(D)** gene-reaction association matrix. In **(A)**, the dashed blue, green, and orange frames indicate the metabolic reactions, enzymes, and genes, respectively. G6P, D-glucose-6-phosphate; F6P, D-fructose-6-phosphate; FDP, D-fructose-1-6-bisphosphate; G3P, glyceraldehyde-3-phosphate; 13DPG, 3-phospho-D-glyceroyl-phosphate; 3PG, 3-phospho-D-glycerate; and Pi, phosphate.

As mentioned above, GEMs are metabolic networks. Figure 1A shows a partly visualized glycolysis pathway in a GEM of *E. coli*, and within this part, we can see that metabolites are linked with each other by reactions, which are associated with enzymes, which are encoded by genes. It should be noted that the stoichiometric coefficient in metabolic reactions in Figure 1A (as shown in Figure 1B) could not be visualized in a graph. Therefore, GEMs employ a stoichiometric matrix (S matrix) to represent all the coefficients in metabolic reactions (Figure 1C). In the S matrix, the *ij*th element represents the stoichiometric coefficient of the *i*th metabolite in the *j*th reaction in the GEM. If the coefficient is positive, the metabolite is produced; otherwise, it's consumed. In addition, the GPR relationships in GEMs are simplified into a two-dimensional binary matrix showing the association between genes and reactions (Figure 1D), in which the *ij*th element is one if the *i*th reaction is associated with the *j*th gene, and it's zero if they aren't associated.

GEMs have several notable features: (1) They are collections of existing knowledge of the metabolism of a specific organism, and in most GEM-based applications, it's assumed that the metabolic network is complete, with very few exceptions, such as for gap finding and gap filling (Latendresse, 2014). (2) They are stoichiometric-balanced networks, which means mass as well as energy balance, reduction, and proton balance are well considered. (3) GPR relationships are annotated in GEMs, but the interactions are not quantitatively described. (4) Even though GEMs describe the metabolism, concentrations of metabolites are not directly included and flux balance analysis (FBA; Orth et al., 2010) is employed for flux simulations, which assumes that there is no (unexpected) accumulation of metabolites within GEMs.

## Using GEMs for essentiality and synthetic lethality analysis

As mentioned above, since GEMs are complete metabolic networks, they can be used for gene/reaction essentiality analysis (EA; Edwards and Palsson, 2000). In general, EA identifies all essential genes or reactions whose knockout will disable a specific biological function through FBA. EA could be easily implemented *in silico* using GEMs by enumerating all single gene/reaction knockouts and testing whether their biological objectives are still functioning. In addition, synthetic lethality analysis (SLA), which scans for combinatory knockouts of multiple reactions/genes that lead to blocking of the target biological function, could also be implemented in a similar way. And recently, several methods have been developed to perform advanced SLA efficiently (Suthers et al., 2009; von Kamp and Klamt, 2014; Pratapa et al., 2015; Zhang et al., 2015).

**Figure 2:**
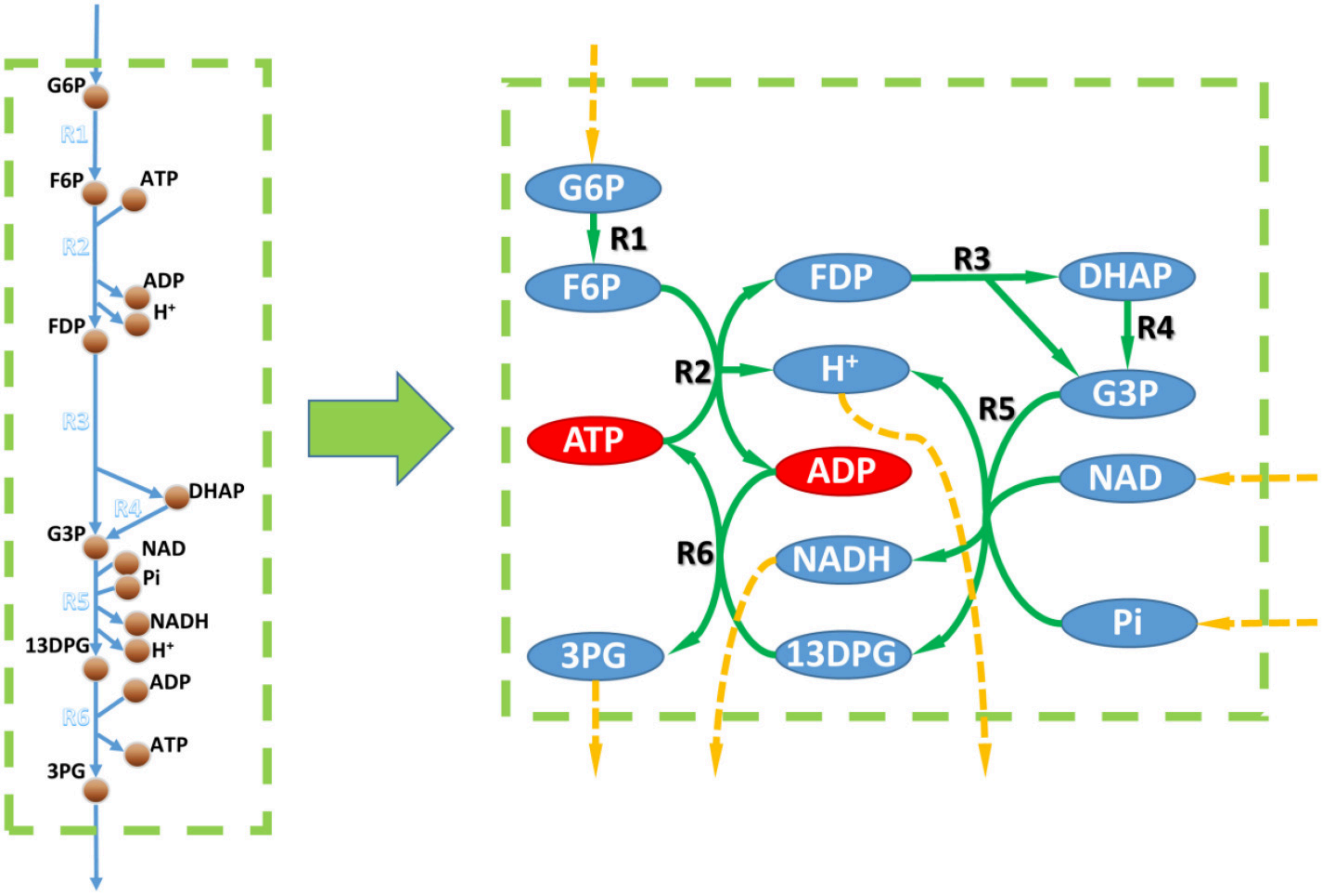
**Metabolic networks vs. GEMs**. Left, metabolic part of the toy model in Figure 1. Right, metabolic network based on the toy model. Circles linked to a dashed orange arrow are unbalanced metabolites within the metabolic network according to topological analysis. Red circles in the right part are metabolites that could not be balanced according to the flux balance analysis based on the toy model.

It's generally believed that gene/reaction EA could be performed by topologic analysis of the metabolic network. However, since the stoichiometric coefficients are absent in topologic metabolic networks, they're less accurate. For example, Figure 2 shows the topologic network of the toy model from Figure 1. Based on its topologic properties, this metabolic work can use D-glucose-6-phosphate, NAD, and phosphate as substrates and produce 3-phospho-D-glycerate, NADH, and a proton. However, this pathway always consumes more ADP than it produces, and produces more ATP than it consumes. Therefore, this pathway will be blocked without ADP supplementation and this finding was not possible by topologic analysis.

Essentially, if a GEM is well established, its EA and SLA results could be very accurate. For example, in the most used *E. coli* and *S. cerevisiae* GEMs, around 90% of the predicted essential genes have been validated *in vivo* (true-negative; Feist et al., 2007; Heavner et al., 2013). This is within expectation, because if a function is blocked *in silico*, it's very unlikely that there could be a complimentary solution *in vivo* to recover it. The explanation for the very few false-negative predictions (negative growth *in silico* and positive growth *in vivo*) is that there's a knowledge gap, such as unknown enzyme or unknown function of an existing enzyme, which leads to the underestimation of the capability of the GEM. On the other hand, even if the GEMs are 100% complete, there may still be false-positive predictions since the missing information of regulation and protein (enzyme) efficiency could lead to extra constraints to GEMs, thereby rendering a nonessential reaction/gene *in silico* essential *in vivo*. It's worth mentioning that, after a certain period of adaptive evolution, a false-positive knockout could become nonessential *in vivo* again (Patil et al., 2005). EA and SLA have mainly been used to validate newly constructed GEMs and in recent years, EA and SLA were applied to study of systems medicine (see Section Using GEMs in Studies of Systems Medicine).

## Using GEMs as scaffolds for multi omics data integration and interpretation

Recently, increasing volumes of transcriptomic, proteomic, and metabolomics data are becoming publically available, and it's believed that GEMs are good scaffolds to make use of these multi omics data. In GEMs, omics data could be quantitatively integrated as constraints for metabolic fluxes, thereby allowing systematic and quantitative evaluation of these data, which was not possible using traditional metabolic networks. This is the most significant advantage of using GEMs as scaffolds.

Although, GEMs are metabolic networks, the most used omic data for GEMs are transcriptomic and proteomic. This is because the technic is really advancing in the field and makes large number of high quality transcriptomic and proteomic data available. However, since the GPR relationships are qualitative in GEMs (Figure 1C), one needs to make assumptions to define the quantitative relationship between gene/protein expression and metabolic fluxes when integrating transcriptomic or proteomic data into GEMs. This is problematic because the complicated relation between fluxes and expression level of genes and enzymes *in vivo* are unlikely to be captured by a general assumption (MacHado and Herrgård, 2014). On the other hand, there're many well-defined approaches to integrate fluxomics and metabolomics, data (Khodayari et al., 2014; Martín et al., 2015; Miskovic et al., 2015). However, it's very difficult (if not impossible) to get genome scale data of them. Hence, we suggest that even though omics data are integrated, one should be skeptical about the quantitative results of simulations or predictions from GEMs.

Nonetheless, we believe that it is better to qualitatively interpret the omics data using GEMs. For instance, it would be much more reliable to use omics data to determine the presence or absence of reactions and to construct high-quality and specific GEMs (Zur et al., 2010; Agren et al., 2012, 2014; Mardinoglu et al., 2013; Yizhak et al., 2014). In addition, many researchers started to integrate significance of differential expression of genes with GEMs rather than their quantitative expression to interpret the biological information behind omic data (Patil and Nielsen, 2005; Cakir et al., 2006; Jensen and Papin, 2011; Fang et al., 2012; Navid and Almaas, 2012). Moreover, qualitative interpretation of omics data with GEMs have recently been applied to systems medicine (see Section Using GEMs in Studies of Systems Medicine). These studies demonstrated the usefulness of GEMs as scaffolds. In short, we suggest that GEMs are powerful platforms for integration of omics data for gaining biological insights rather than quantitative results.

## Using GEMs for *in silico* metabolic engineering

Using GEMs for *in silico* metabolic engineering has been a widely discussed topic for years. It's generally believed that GEM-based methods could predict gene modification strategies for overproduction of desired biochemicals and thus, accelerate the overall metabolic engineering process. In the last decade, various kinds of *in silico* metabolic engineering methods had been developed and many of them were applied experimentally (Kim et al., 2015; Long et al., 2015; MacHado and Herrgård, 2015).

Although *in silico* metabolic engineering methods seemed quite different from each other, they follow a similar procedure: (1) they define what a desired strain is and (2) identify approaches that push the wild-type strain to become the desired one. So far, a variety of approaches were used in *in silico* metabolic engineering, such as reaction/gene knock-out (Burgard et al., 2003; Patil et al., 2005; Kim et al., 2012; Ren et al., 2013; Ruckerbauer et al., 2014; Zhang et al., 2015), overexpression/suppression (Pharkya and Maranas, 2006; Choi et al., 2010; Ranganathan et al., 2010; Park et al., 2012; Chowdhury et al., 2014), foreign pathway knock-in (Pharkya et al., 2004), and swapping the co-factor for a target enzyme (NADH to NADPH or vice versa; King and Feist, 2013). However, the methods for knock-out identification are the majority since a knockout is much easier to define *in silico* than up-/down-regulation of genes as mentioned before. On the other hand, different methods could have independent definition of desired strains. For instance, some of the methods define the desired strain by simply setting thresholds for growth and production, respectively, and others could define the desired strain following some biological assumptions (Edwards et al., 2001; Segrè et al., 2002).

Interestingly, methods pursuing different type of desired strains could all lead to experimentally valid strategies for metabolic engineering (Fong et al., 2005; Trinh et al., 2008; Fowler et al., 2009; Choi et al., 2010; Yim et al., 2011; Ng et al., 2012; Nocon et al., 2014), but the production of target products predicted *in silico* seldom achieved *in vivo*. The explanation to this is complicated, and could come from both the computational and experimental side. However, one of the key reasons should be that GEM with only metabolic network is not enough to quantitatively predict the behavior of strains *in vivo*. In conclusion, we suggested that all kinds of *in silico* metabolic engineering methods are instructive, but it's better to use them for gaining information rather than to develop exact strategies.

## Using GEMs in studies of systems medicine

Using GEMs for systems medicine studies have recently been highlighted (Mardinoglu and Nielsen, 2015; Yizhak et al., 2015). GEMs simulate the human metabolism in a holistic way, and this greatly advances systems medicine studies by enabling systematic evaluation of metabolic feature of human disease. Great efforts had been made in reconstructing GEMs of human, and there're now several publically available generic human metabolic networks such as Recon 1, Recon 2, EHMN, and HMR (Duarte et al., 2007; Ma et al., 2007; Agren et al., 2012; Thiele et al., 2013). In addition, since the technology is advancing, tissue specific or cell specific genomic, proteomic and transcriptomic data are becoming available (Cancer Genome Atlas Research Network, 2008; Uhlén et al., 2015). These led to rapid development in reconstruction of high quality tissue or cancer specific GEMs (Zur et al., 2010; Agren et al., 2012, 2014; Mardinoglu et al., 2013) and, therefore, enabled more confident interpretation of metabolism of diseases.

For instance, cancer specific GEMs together with EA and SLA analysis were recently used for identification of oncogenes/metabolites and biomarkers for diagnosing specific cancer (Agren et al., 2014; Jerby-Arnon et al., 2014; Gatto et al., 2015; Gatto and Nielsen, 2015). Since this procedure mainly uses the true-negative part of EA and SLA, the analysis could be highly reliable. For example, (Agren et al., 2014) identified 101 drug targets for liver cancer treatment; and 83 of them are currently in use or have shown strong correlation with cancer progression. In addition, together with multi-omic data, GEMs were used to find the mechanistic explanation of various diseases. By interpreting clinical omic data with GEMs, the mechanistic understanding of non-alcoholic fat liver disease and type two diabetes were reported (Mardinoglu et al., 2014; Väremo et al., 2015). Moreover, GEMs were also used to explore the effect of microbiota (Ji and Nielsen, 2015). By simulate and predict the interaction of gut microbiota and their effect on hosts, several recent studies revealed that microbiota modulate the amino acid and glutathione metabolism of their host (Shoaie et al., 2013, 2015; Mardinoglu et al., 2015). These exciting studies exhibited the great potential of GEMs in the field of systems medicine, and hopefully there would be much more excellent works coming out.

## Discussion

GEMs are very useful platforms and tools for systems biology, but they're still very young compared to traditional ones. Fluxes of reactions could be quantitatively simulated using GEMs, although caution should be exercised before drawing conclusions based on simulated fluxes owing to the huge solution space of GEMs (Reed, 2012). Although solution space could be reduced by adding constraints through integration of omics data, it would be better to gain biological insights by qualitative interpretation of omics data rather than quantitative fluxes.

In order to achieve accurate quantitative prediction, the scope of GEMs should be expanded. The establishment of ME-models set a good example for this (Thiele et al., 2009, 2012). In ME-models, the interaction of genes (mRNA), enzymes, and metabolic fluxes are quantitatively expressed, enabling proper integration of transcriptomic and proteomic data. However, it is still difficult to integrate metabolomics data into ME-models. A potential option to integrate metabolite concentration into GEMs is cybernetic modeling. However, to date, there has been no study on genome-scale cybernetic modeling because there are too many parameters to simulate, making it computationally infeasible.

In general, no model is perfect. Genome-scale modeling methods are still under development and have several drawbacks. In addition, it has been recently reported that many published GEMs are of low qualities (Chindelevitch et al., 2014; Ravikrishnan and Raman, 2015). Therefore, they should be used with caution. As concluded in this review, GEMs are more suitable for qualitative applications at this stage, such as EA and SLA analysis. When using GEMs for quantitative applications such as *in silico* metabolic engineering, one should be aware of the key assumption behind the method and take the results as instructions. However, it should also be noted that, GEMs are open platforms and have great potential in a wide array of applications. Currently, GEMs are used for simulating the interactions between multiple organisms, multiple tissues (Bordbar et al., 2011), and even between microbiota and human tissues. On the other hand, EA and SLA were developed years ago, but they were not used in the discovery of anti-cancer drugs until recent years. These are good examples of how to explore novel applications based on classical methods. Thus, in future, GEMs can be expected to be more widely used in biotechnology, bioengineering, and many other fields.

## Author contributions

CZ conducted the writing of this paper, and QH modified and edited it.

### Conflict of interest statement

The authors declare that the research was conducted in the absence of any commercial or financial relationships that could be construed as a potential conflict of interest.
